# Understanding how general practice nurses support adult lifestyle risk reduction: An integrative review

**DOI:** 10.1111/jan.15344

**Published:** 2022-06-30

**Authors:** Maksi Morris, Elizabeth Halcomb, Yazdan Mansourian, Maree Bernoth

**Affiliations:** ^1^ School of Nursing, Paramedicine and Healthcare Sciences, Faculty of Science Charles Sturt University Wagga Wagga NSW Australia; ^2^ School of Nursing, Faculty of Science, Medicine & Health University of Wollongong, Illawarra Health & Medical Research Institute Wollongong NSW Australia; ^3^ School of Information and Communication Studies, Faculty of Arts and Education Charles Sturt University Wagga Wagga NSW Australia

**Keywords:** behaviour modification, general practice, health promotion, integrative review, lifestyle change, nursing, office nursing, prevention, primary care, risk reduction

## Abstract

**Aim:**

To review the literature exploring how general practice nurses support lifestyle risk reduction.

**Design:**

Integrative literature review.

**Sources:**

CINAHL, Emcare, MEDLINE, Proquest and Scopus were searched for peer‐reviewed primary research published in English from 2010 to 2022.

**Methods:**

Sixteen papers met the inclusion criteria and were assessed for methodological quality using the Mixed Methods Appraisal Tool. Findings were extracted and thematically analysed.

**Results:**

Four themes described general practice nurses: (1) Establishing relational connections; (2) Empowering active participation; (3) Engaging mutual motivation and (4) Enabling confident action. General practice nurses used complex interpersonal, risk communication and health coaching skills to build collaborative partnerships that supported patients' self‐determination and self‐efficacy. While mutual motivation and confidence were reciprocally enabling, gaps in skills, experience and knowledge plus time, resource and role constraints limited general practice nurses' ability to support lifestyle risk reduction.

**Conclusion:**

General practice nurses play a key role in lifestyle risk reduction. Ongoing education, funding, organizational and professional support are needed to enhance their commitment, confidence and capacity.

**Impact:**

- **What problem did the study address?**
  While general practice nurses play a key role in health promotion and risk reduction, their potential is yet to be fully realized. Research examining methods by which nurses working in general practice support lifestyle risk reduction is limited.
- **What were the main findings?**
  Successful interactions depended on personal, professional, organisational and systemic factors which either enhanced or inhibited relational quality, shared decision‐making, mutual commitment, and nurses' confidence and capacity to address lifestyle risks. Targeted professional development and peer mentoring are needed to build proficient practice.
- **Where and on whom will the research have impact?**
  Understanding how general practice nurses support risk reduction can inform policy and identify training and support needs to advance their skills and role. Research exploring synergies between themes may illuminate this process.

## 1 INTRODUCTION

Lifestyle changes caused by globalization and urbanization, population ageing, socio‐economic inequity and convenience food marketing have driven a surge in chronic conditions worldwide. Leading causes of premature mortality, morbidity and disability, the World Health Organization (WHO) (2020) reports that 74% of deaths in 2019 and 62% of the 2018 total disease burden were attributed to chronic illness. Chronic ill health reduces quality of life, drives demand for health services, and inflates the cost of health care (WHO, 2019). Fortunately, many chronic conditions are preventable, sharing modifiable behavioural risk factors including tobacco use, unhealthy diet, harmful use of alcohol and physical inactivity (WHO, 2019, 2020). In 2019, 7.1 million deaths worldwide were related to tobacco use, 10.1 million to diet, 2.84 million to alcohol use and 1.26 million to physical inactivity (Ritchie & Roser, 2018). While a raft of prevention and control initiatives have reduced smoking rates, tobacco use is still a leading cause of illness. Meanwhile, progress on dietary risk and inactivity has been limited and inaction on alcohol remains widespread (Department of Health, 2021; WHO, 2020).

Approximately 80% of Australia's burden of disease is attributed to chronic illness, 38% of which may be prevented by addressing lifestyle risks (Bartlett et al., 2016). Enhancing health literacy, promoting healthy behaviours and facilitating risk reduction can prevent, delay and slow the progression of many chronic conditions (Royal College of General Practitioners [RACGP], 2016). The benefits of risk screening, targeted health education, brief interventions, lifestyle prescription, behavioural counselling and referrals generated by primary care providers have been established (Department of Health, 2021). Nevertheless, historic underinvestment, fragmented policy, leadership inadequacies and focus on treatment have been persistent barriers thus clinical support for lifestyle risk reduction remains suboptimal and inconsistently applied in usual care (James et al., 2018). Renewed policy directions propose greater investment to improve social determinates of health, boost action to reduce lifestyle risks, and enable the health workforce to work to their full scope to promote health and prevent illness through multidisciplinary care (Department of Health, 2021). Greater adoption of primary health care approaches (WHO, 2020) including increased availability of primary care services and enhanced capacity and skills of health professionals is key to advancing this agenda (Department of Health, 2018).

### 1.1 Background

Over 80% of Australians see a general practitioner (GP) at least annually, thus primary care is an important setting that provides opportunities to identify lifestyle risk and support behaviour change that enhances health outcomes (Halcomb et al., 2015). Changing patients risk behaviours requires sustained strategies including individual assessment, patient education and health coaching, goal setting, referral and follow‐up (Harris et al., 2017; RACGP, 2015). However, workforce shortages, system inefficiencies, maldistribution of services, growing health inequities and demand for chronic disease management (CDM), particularly in rural areas, continue to constrain capacity for preventive health activities (Department of Health, 2021). Like the United Kingdom, Europe and New Zealand, Australia has increased the numbers of general practice nurses (GPNs) to support the medical workforce and increase access to primary care services (James et al., 2018). With an estimated 63% of general practices employing a nurse, general practice nursing is currently the fastest growing area of the health system. Comprising mainly baccalaureate‐trained registered nurses, general practice nursing falls within the domain of primary health care. These nurses have clinical roles in population health, health promotion, disease prevention, risk factor screening and CDM (Australian Primary Health Care Nurses Association, 2018; Halcomb et al., 2017).

Nurses working in collaboration with GPs can increase the capacity for preventive care in general practice by facilitating risk assessment, promoting risk reduction strategies through health education and self‐management and supported behaviour change (Halcomb et al., 2014; Harris et al., 2017). Nurses spend more time with patients than GPs, communicate more effectively and elicit greater engagement with clinical care. Regular contact with at‐risk patients enables GPNs to establish relationships that facilitate lifestyle risk communication (James et al., 2018). Clinically and economically effective and sustainable interventions provided by GPNs that facilitate risk reduction, build health literacy and promote self‐management are acceptable to patients and clinicians (James et al., 2018). Even so, role ambiguity, lack of career framework, insufficient pre‐clinical preparation and experience, and inadequate organizational support continue to limit the expansion and optimization of the GPN role (Desborough et al., 2018; James et al., 2018).

Opportunities exist to strengthen and advance GPNs' role in health promotion and prevention, especially in rural and remote areas where the prevalence of chronic illness and lifestyle risks is greatest (Halcomb et al., 2017). To date, preventive research has focused on interventions targeting specific conditions or single risk factors. Further research is needed to understand how preventive interventions may be more broadly and effectively implemented in routine primary care (Marks et al., 2020). Exploring how GPNs' currently support lifestyle risk reduction can furnish insights to inform policy and curriculum developments and strengthen organizational supports aimed at optimizing the GPNs' role and advancing best practice.

## 2 THE REVIEW

### 2.1 Aims

This integrative review aims to explore how GPNs support adult patients to reduce lifestyle risks associated with chronic disease.

### 2.2 Design

An integrative review was chosen to allow the synthesis of papers reporting diverse research methodologies. The approach described by Whittemore and Knafl (2005) was applied through stages of problem identification, literature search and evaluation and data analysis and presentation. The 2020 guidelines for the Preferred Reporting Items for Systematic Reviews and Meta‐Analyses (PRISMA) (Page et al., 2021) guided reporting of this review.

### 2.3 Search methods

**FIGURE 1:**
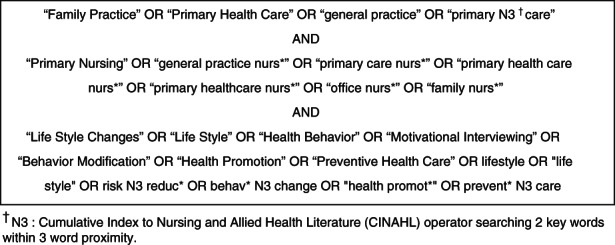
Search terms

The search strategy involving keyword searching of CINAHL, Emcare, MEDLINE, ProQuest and Scopus databases (Figure 1) for peer‐reviewed primary research papers published in English from January 2010 to February 2022. Papers reporting GPNs' (registered nurses) interactions with adults to reduce lifestyle risks were included. Due to differences in scope of practice, papers focused on nurse practitioners, specialists and enrolled nurses were excluded (Table 1). Due to resource constraints and the likelihood that peer‐reviewed papers would be more robust, the grey literature was not included in the search strategy. Hand searching of the reference lists of included papers and key journals yielded no additional papers.

**TABLE 1:** Inclusion and exclusion criteria

Inclusion criteria	Exclusion criteria
GPN interactions with adults to support behaviour change to reduce lifestyle risks. Original research published January 2010 to February 2022. Published in a peer‐reviewed journal in English language.	Unable to isolate or extract data on GPN support to reduce lifestyle risks among adults. Focus on GPN perspectives of an intervention study. Focus on nurse practitioners, specialist practice, and enrolled nurses, general practitioners and allied health professionals. Discussion papers, editorials and literature syntheses.

Abbreviation: GPN, General practice nurse.

### 2.4 Search outcomes

**FIGURE 2:**
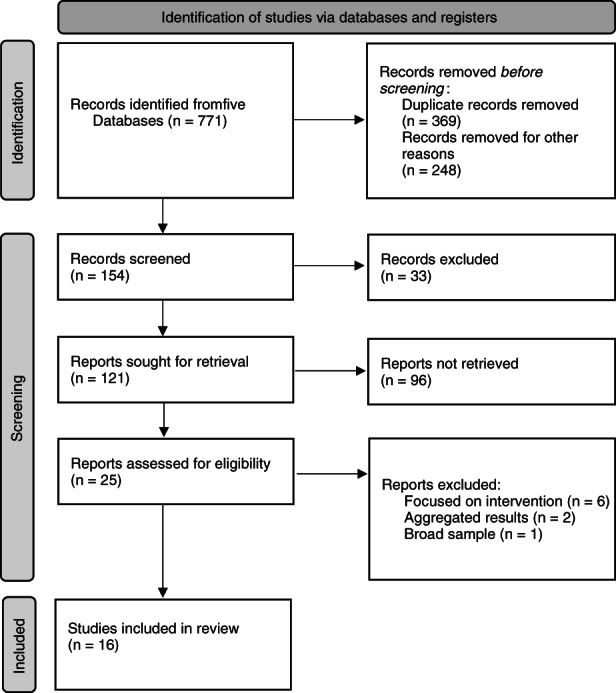
PRISMA selection process.

Database searching identified 771 potentially relevant records from CINAHL, Emcare, Proquest; Medline; and Scopus databases which were exported into EndNote X9 (The Endnote Team, 2013). Following the removal of 369 duplicates and 248 non‐original research and 33 non‐relevant papers, the titles and abstracts of 121 papers were reviewed against the inclusion/exclusion criteria by two authors (MM and EH). Twenty‐five full‐text papers were then independently screened by all authors, 16 of these met the inclusion criteria (Figure 2).

### 2.5 Quality appraisal

Due to the diversity of research approaches, the Mixed Methods Appraisal Tool (MMAT) was used to assess methodological quality (Hong et al., 2018). Papers were independently evaluated by two authors (MM and YM). Scores were compared, discrepancies were discussed and a consensus was reached (Appendix S1). Eleven papers scored 100% (Aranda & McGreevy, 2014; Beishuizen et al., 2019; Bräutigam Ewe et al., 2021; Geense et al., 2013; Hornsten et al., 2014; James et al., 2020a, 2020b; James et al., 2021; Jansink et al., 2010; Keleher & Parker, 2013; Phillips et al., 2014), one scored 80% (Tong et al., 2021), and four scored 60% (Goodman et al., 2011; McIlfatrick et al., 2014; Walters et al., 2012; Westland et al., 2018). Due to relatively minor methodological flaws and small number of included papers all 16 papers were retained (Whittemore & Knafl, 2005).

### 2.6 Data abstraction and synthesis

Findings from included papers were abstracted into a summary table to facilitate comparison (Table 2). Following methods described by Braun and Clarke (2006), inductive analysis involved immersion in the data to identify patterns and relationships between them across the data set. Initially, recurring concepts were coded and then collated into preliminary themes which were reviewed, defined and labelled. Analysis and synthesis of refined themes were verified by all authors (Whittemore & Knafl, 2005).

## 3 RESULTS

### 3.1 Included papers

Sixteen papers described 12 studies from six countries, with one study producing three papers (James et al., 2020a, 2020b; James et al., 2021) (Table 2). Most papers were from Australia (*n* = 6; 37.5%) and the Netherlands (*n* = 4; 25%), which included one joint paper from the Netherlands and Finland. The majority of papers were qualitative (*n* = 10; 62.5%) and interviews were the most common method of data collection (*n* = 9; 56.3%). Six papers (37.5%) discussed the prevention of multiple risk factors (Beishuizen et al., 2019; Geense et al., 2013; James et al., 2020a, 2020b; James et al., 2021; McIlfatrick et al., 2014), five reported on a single lifestyle risk factor (Aranda & McGreevy, 2014; Bräutigam Ewe et al., 2021; Goodman et al., 2011; Phillips et al., 2014; Tong et al., 2021), and two explored health promotion and prevention (Geense et al., 2013; Keleher & Parker, 2013). Also, three papers examined interactions related to specific chronic conditions (Jansink et al., 2010; Walters et al., 2012; Westland et al., 2018). Four themes, discussed below, describe how GPNs support lifestyle risk reduction by (1) Establishing relational connections; (2) Empowering active participation; (3) Engaging mutual motivation and (4) Enabling confident action.

**TABLE 2:** Summary of included papers

Author/Country	Aim	Sample	Methods	Findings
Aranda and McGreevy (2014) England	To explore perceptions & experiences of overweight GPNs on obesity management	7 GPNs	Qualitative Interviews	Personal experience provided insights into patients emotional struggles, social stigma, factors contributing to weight gain and barriers to weight loss. GPNs^a^ were conscious of how their weight impacted professional credibility; were sensitive about initiating discussions and drew on personal understandings and experiences rather than strictly adhering to guidelines.
Beishuizen et al. (2019) Netherland & Finland	To explore GPNs best practices in behaviour change support for integration into an online cardiac risk prevention platform	13 GPNs	Qualitative Focus groups	GPNs viewed their role as supporting patient‐led lifestyle change and identified three preconditions for supporting patients accessing an online cardiac risk prevention tool: Establishing trusting relationships and providing individually tailored support. Raising awareness of personal risk; stimulating motivation through education & coaching; managing unrealistic expectations; and preparing for challenges. Appropriate timing and monitoring via regular GPN follow‐up to promote adherence.
Bräutigam Ewe et al. (2021) Sweden	To describe GPNs general perceptions of overweight, experiences of overweight /obesity in clinical practice, & visions for working with lifestyle issues	13 GPNs	Qualitative Interviews	GPNs indicated that schools and mass media; regulation of the food market; and health promotion beyond healthcare objectives were arenas for expanding HP at a societal level. GPNs reported that positive interactions; tailored interventions; and collaborative care were conducive to lifestyle change. However, uncertainty about implementing guidelines plus ethical and cultural issues were challenges. GPNs perceived that the degree of patient motivation determined outcomes. While motivating resistant patients was difficult, positive results were professionally rewarding. GPNs stated that patients were responsible for their health choices and that parents needed to role model health behaviours. Nevertheless, GPNs recognized the impacts of education, health literacy, SES.
Geense et al. (2013) Netherlands	To explore clinicians current health promotion activities, attitudes, main topics plus barriers and enablers of health promotion activities	16 GPs 9 GPNs	Qualitative Interviews	GPNs often gave smoking cessation and dietary advice while GPs^b^ advised about alcohol reduction. Barriers and facilitators related to (1) patients; (2) practitioners/practice settings; (3) provider attitudes; (4) programmes and (5) health care systems/government policy. Barriers: (1) comorbidities; low SES^c^ and health literacy; and complexity of behaviour change. (2) Lack of skill, time, interdisciplinary collaboration, facilities, and referral options; difficult to measure results; incongruent personal behaviour and professional roles; demotivation due to disappointing results. (3) Perception that patients are unwilling; low priority; focus on treatment; scepticism. (4) Lack of availability, accessibility, proven efficacy and discontinuity of local programmes. (5) Lack of funding for programmes and GPN time; changing subsidies and requirements; inconsistent policy; and poor intersectoral cooperation. Facilitators: (1) Awareness of risk and motivation for change; patient‐led agenda. (2) Optimizing GPN role; availability and time; co‐located allied health services. (3) Lifestyle risk reduction perceived worthwhile and part of GPNs role; (4) Familiarity with patients; easy access and affordability; (5) Clear policy; incentivized participation.
Goodman et al. (2011) England	To determine the current level of GPN involvement, knowledge & attitudes toward activity promotion for older adults	391 GPNs	Quantitative Surveys	GPNs encouraged physical activity by assessing current activity; providing advice; suggesting activities; and referring patients to specialist services. GPNs were confident to provide appropriate activity advice to uncomplicated sedentary patients but not to patients with chronic conditions. Lack of time (88%) and training (58%); poor staffing; organizational constraints; referral problems; and intermittent patient contact were barriers. There was no association between GPN activity levels and activity promotion in older adults.
Hornsten et al. (2014) Sweden	To describe communication strategies used by GPNs in health‐promoting dialogues	10 GPNs	Qualitative Interviews	Five contrasting themes described GPNs communication strategies. Guiding versus Pressuring: GPNs highlighted issues and offered tailored solutions or focused on problems and demanded change. Adjusting to the patient versus Directing the conversation: GPNs either used flexible approaches and were patient‐centred or prioritized their professional agenda. Inspiring confidence versus Instilling fear: GPNs either encouraged positive choices or emphasized the severity of risks and potential illness. Motivating and supporting versus Demanding responsibility: Some GPNs overcame resistance; made change achievable; increased patient self‐efficacy while others provided information about results; and placed an onus on patients to effect change. Introducing emotive topics versus Avoiding. Some GPNs were confident to initiate discussions and used training and experience to meet resistance. Others avoided hard to raise topics, people or issues.
James et al., (2020a, 2020b) Australia	To examine what communication skills GPNs used & how these are employed to reduce lifestyle risk	14 GPNs	Mixed Methods Video observations	GPNs explored lifestyle risk by informally building rapport; determined the agenda by assessing lifestyle behaviours, risk factors or following GP referral; affirmed and encouraged healthy choices; clarified priorities; and confirmed understanding using reflective listening. Opportunities for further exploration, agenda‐setting and education were sometimes missed; confidence & importance levels were not assessed; GPNs often did not summarize patient priorities. When they occurred, discussions related to patient choice, goal setting and action planning were prolonged. Closed questions and statements were sometimes used to present options. Reflective, affirming statements were used to show empathy for barriers to change.
James et al., (2020a, 2020b) Australia	To explore GPNs perceptions of interactional factors that support lifestyle risk communication.	15 GPNs	Mixed Methods Interviews	Communication ranged from patient‐led to the use of scare tactics. Most GPNs adapted their communication style and information according to patient needs and capacity. Approachability and relational continuity helped GPNs establish rapport, trust and familiarity necessary for ongoing, open dialogue. Successful discussions depended on the patients motivation, readiness and capacity to prioritize lifestyle change. Some GPNs initiated discussions and addressed barriers to change whereas others responded only when patients indicated readiness to change. Patient lack of awareness of the GPN role led to misconceptions that reduced the duration and content of lifestyle communication.
James et al. (2021) Australia	To explore barriers & facilitators to GPNs lifestyle risk communication	15 GPNs	Mixed Methods Interviews	Barriers: (1) educational preparation, professional development and confidence affected GPNs engagement in lifestyle risk communication; (2) organizational practices, limited time and funding arrangements constrained the prioritization of lifestyle risk communication; (3) lifestyle risk prioritization was considered less relevant than other clinical tasks or patient needs. Facilitators: (1) organizational support including the availability of time, space, resources and interprofessional collaboration created opportunities for lifestyle risk communication; (2) autonomous roles, enhanced by GPNs education, experience, confidence and accountability, supported lifestyle risk communication; (3) supporting patients needs and main concerns supported lifestyle risk reduction.
Jansink et al. (2010) Netherlands	To examine barriers to lifestyle counselling of diabetic patients to inform an intervention study	13 GPNs	Qualitative Interviews	GPNs felt they had insufficient knowledge to provide diet and physical activity counselling, were unmotivated and considered lifestyle counselling ineffective. GPNs had difficulty adapting communication; resisting directive approaches; maintaining appropriate expectations; developing action plans; and involving patients in decision‐making. GPNs believed patients lacked insight and knowledge about their health; made excuses; and were noncompliant. Low literacy and SES; social and cultural influences; addiction and relapse; and psychological issues were perceived barriers for patients. Lack of time; understanding of roles; collaborative practice; & knowledge of local resources were organizational barriers.
Keleher and Parker (2013) Australia	To explore GPNs perceptions of current & potential roles in health promotion (HP)	54 GPNs	Qualitative Surveys	GPNs described HP in the context of established roles in chronic disease management. Often opportunistic, HP activities involved patient education and brief interventions. Most described a potential to work beyond the management practices that defined their role and directed their work. Opportunities for expanded roles in HP included conducting lifestyle clinics and groups sessions; implementing recalls and reviews; delivering smoking cessation programmes; building therapeutic relationships; and facilitating multidisciplinary care. Funding structures and GP support were important enablers of GPN role expansion. Resistance to expanding roles; inadequate knowledge, skills, time and appropriate space; poor organizational capacity and interprofessional collaboration were common constraints.
McIlfatrick et al. (2014) Ireland	To examine clinicians current and potential roles, explore facilitators/barriers & identify strategies to overcome difficulties in cancer prevention	Survey 225 GPNs Interview 15 GPNs	Mixed Methods Surveys Interviews	GPNs formed 15% of the survey sample and 14% had completed post‐graduate training in cancer prevention/treatment. Financial incentives focused preventive services on smoking and cervical screening (both 96.3%), obesity (94.5%), physical activity (84.9%), diet (82.5%) and alcohol (79.4%). GPNs most often provided information leaflets (84.3%) and brief advice (83.0%) for smoking cessation; asked patients about activity levels (84.9%) and diet (82.5%); provided weight management information (51.2%) literature about diet (46.1%). Most believed they could motivate patients (99.5%); that patients accepted their role (80%); were receptive to change (60.4%); and would follow advice (78.9%). Only 58.7% felt sufficiently knowledgeable to provide education and 84.1% wanted further training in effective behaviour change methods. Interviewed GPNs stressed the need to gain trust; develop therapeutic relationships over time; & follow the patients agenda. Challenges included feeling ineffective, patient ambivalence and disadvantage.
Phillips et al. (2014) Australia	To explore how GPNs manage obesity to identify good practice & barriers to effective management	18 GPNs	Qualitative Interviews	GPNs routinely provided weight advice to patients who attended for CDM, were newly diagnosed or presented with weight‐related problems. Opportunistic weight discussions were thought to alienate patients and endanger therapeutic relationships. Familiar therapeutic relationships facilitated openness and honesty. Personal experiences were used to demonstrate empathy. GPNs were confident to assess patient readiness for change and motivational interviewing strategies were used to assess patients motivation, expectations and confidence. Self‐esteem and self‐image were explored, immediate benefits of lifestyle change were highlighted, and goals were linked to relevant life events. GPNs provided individually tailored dietary advice; promoted activity patients enjoyed; tailored suggestions to address barriers; and emphasized small, sustainable changes increasing in intensity over time. Self‐monitoring options and smart technology were sometimes suggested. While GPNs encouraged patient‐led behaviour change; low health literacy; unrealistic expectations; complexity of change; and GPNs lack of confidence were barriers.
Tong et al. (2021) England	To describe elements of GPNs consults for weight loss, behaviour change techniques (BCTs) & dietary/physical activity recommendations	8 GPNs	Quantitative Audiotaped consultations	Content analysis of 51 audio recorded GPN consults with overweight / obese patients. The 93‐point BCT tool was used to assess use of BCT. GPN consults per patient averaged 2.8, the mean duration per patient over a 3mth period was 36.9 mins. Weight changes measured at 3, 6 and 12 months were − 3.6% (3.5 kg), −5.5% (5.5 kg), & ‐4.2% (4.0 kg) respectively. 29/93 BCT were used at least once; 3.9 BCT were used per consult per patient; and 10.6 BCT were used per patient overall. GPNs used BCT ‘feedback on behaviour’ (80.0%); ‘problem solving’ (38.0%) and ‘social reward’ (34.3%) most often. 24/30 dietary recommendations were used, most often ‘portion size’ (31.3%). 9/10 physical recommendations were used, most often ‘encouragement of walking’ (30,3%). Longer consults and No. BCT used were positively associated. There was no significant correlation between the average No. of BCTs / diet and activity recommendations per consult and weight change.
Walters et al. (2012) Australia	To investigate potential roles for GPNs in health mentoring (HM) for chronic disease self‐management	5 GPNs	Mixed Methods Surveys Interviews	GPNs were surveyed prior and interviewed following HM training. Pre‐training: GPNs indicated a high degree of role engagement, autonomy, collegiality, organizational support and collaboration. Lifestyle advice was usually provided during consults for CDM and less often in routine care. The importance of respecting patient preferences and working in partnership was recognized. Nevertheless, GPNs said they generally told patients what to do. GPNs confidence in setting health goals, developing action plans and mentoring patients through difficulties was mixed. Post‐training: GPNs wanted HM approaches embedded in routine care and recognized a need for frequent, regular contact however heavy workload and low task priority were barriers.
Westland et al. (2018) Netherland	To examine self‐management topics, duration and frequency & behaviour change techniques (BCT) used by GPNs to support self‐management	17 GPNs	Quantitative Audiotaped consultations	Content analysis of 78 routine GPN consults with patients diagnosed with chronic conditions was measured against 49 health and self‐management topics (H/SM). The 93 point BCT tool was used to assess the application of BCT. GPNs briefly addressed H/SM topics including diet (76.9%); physical activity (71.8%); understanding the disease (65.4%); exacerbation management (61.5%); medication management (57.7%); symptom monitoring (41%); alcohol use (37.2%); and smoking cessation (34.6%). BCT were applied implicitly. Most GPNs (n = 11) ‘reviewed behaviour goal(s)’ and ‘gave feedback on behaviour’ most often; the majority (n = 13) ‘gave information about health consequences’ least often. GPNs rarely assisted with goal setting and action planning. While barriers to change were discussed, strategies for overcoming them were not explored.

^a^ GPN, General practice nurse.

^b^ GP, General practitioner.

^c^ SES, Socio‐economic status.

### 3.2 Establishing relational connections

Establishing cooperative relationships was considered foundational to risk reduction interventions and was, therefore, a central concern in many papers (Beishuizen et al., 2019; Bräutigam Ewe et al., 2021; Hornsten et al., 2014; James et al., 2020b; Keleher & Parker, 2013; McIlfatrick et al., 2014; Phillips et al., 2014). Formed through joint participation, and based on mutual trust, respect and understanding, nurse–patient relationships created opportunities to address lifestyle risks and support behaviour change (Hornsten et al., 2014; James et al., 2020b; McIlfatrick et al., 2014). The significance of reciprocal relationships was confirmed by Westland et al. (2018), who observed interactions often involve sharing of patients' and nurses' personal lives. Relational continuity allowed mutually satisfying relationships to develop, deepening nurses' knowledge of patients' life contexts, priorities and preferences (Beishuizen et al., 2019; Hornsten et al., 2014; James et al., 2020b; Phillips et al., 2014). While Keleher and Parker (2013) showed that GPNs wanted to cultivate relationships to facilitate health promotion, some papers described nurses' reluctance to broach lifestyle topics for fear of jeopardizing therapeutic relationships (Jansink et al., 2010; Phillips et al., 2014).

Importantly, empathy facilitated openness, trust and understanding; creating an environment of psychological safety that ameliorated potentially emotionally charged discussions (Aranda & McGreevy, 2014; Beishuizen et al., 2019; Bräutigam Ewe et al., 2021; James et al., 2020b; Phillips et al., 2014). While GPNs' ability to demonstrate empathy developed with practice and experience (Aranda & McGreevy, 2014; Hornsten et al., 2014), their appreciation for the complexity and challenges of risk reduction in the context of chronic conditions, advancing age, disadvantage and cultural diversity was varied (Bräutigam Ewe et al., 2021; Hornsten et al., 2014; Jansink et al., 2010; Phillips et al., 2014). Some GPNs used reflective, affirming statements (James et al., 2020b), self‐disclosure, and shared experiences of lifestyle challenges (Aranda & McGreevy, 2014; Phillips et al., 2014) to demonstrate empathy and cultivate caring relationships. Those without lived experience showed less empathy for patients' challenges (Jansink et al., 2010). Dissonance between nurses' personal attitudes, lifestyle behaviours and professional roles sometimes led to relational distancing and avoidance of lifestyle topics (Aranda & McGreevy, 2014; Geense et al., 2013; Jansink et al., 2010).

### 3.3 Empowering active participation

Participatory approaches that demonstrated respect for patient autonomy and personal choice encouraged shared decision‐making which, in turn, strengthened motivation and capacity for change (Hornsten et al., 2014; James et al., 2020a). While objectives were sometimes directed by general practitioner referral and nurses' priorities (James et al., 2020a; Jansink et al., 2010), empowering patients to negotiate their own agenda and goals were understood to increase the success of interactions (Geense et al., 2013; James et al., 2021; Phillips et al., 2014). Several studies related the need to adapt communication and tailor support to suit patients' unique context, capacity and agenda (Bräutigam Ewe et al., 2021; Hornsten et al., 2014; James et al., 2020a; Walters et al., 2012; Westland et al., 2018). Bräutigam Ewe et al. (2021) highlighted the importance of adapting communication and support to patients' life contexts to achieve sustainable results. However, some nurses had difficulty adjusting their expectations and approach to accommodate the patients' readiness for change and to facilitate shared decision‐making (Hornsten et al., 2014; James et al., 2020a; Jansink et al., 2010). Directive approaches were adopted with patients who were considered non‐compliant, resistant or indifferent (Aranda & McGreevy, 2014; Geense et al., 2013; Hornsten et al., 2014; James et al., 2020b; Phillips et al., 2014).

Social and emotional sensitivity and mindful communication also strengthened partnerships and enhanced shared decision‐making (Aranda & McGreevy, 2014; Bräutigam Ewe et al., 2021; Hornsten et al., 2014; James et al., 2020b). By conveying approachability, warmth, respect and non‐judgement, nurses fostered an atmosphere of receptivity and psychological safety that facilitated patient participation (Aranda & McGreevy, 2014; Beishuizen et al., 2019; Hornsten et al., 2014; James et al., 2020b; McIlfatrick et al., 2014; Phillips et al., 2014). James et al. (2020b) observed affirming and encouraging statements that reinforced positive lifestyle choices and increase motivation. Participants described using active listening, open questioning and reflective feedback to clarify patients' priorities, determine readiness for change, discuss change options, negotiate goals and encourage shared decision‐making; however, these skills were often inconsistently applied (Hornsten et al., 2014; James et al., 2020a; Jansink et al., 2010; McIlfatrick et al., 2014; Phillips et al., 2014; Westland et al., 2018).

General practice nurses generally acknowledged their professional obligation to address lifestyle risks (Bräutigam Ewe et al., 2021; Goodman et al., 2011; McIlfatrick et al., 2014; Phillips et al., 2014; Walters et al., 2012). Some described playing a central role in lifestyle risk reduction, proactively promoting healthy lifestyles and improving patients' attitudes toward making lifestyle changes to reduce risk and prevent illness (Geense et al., 2013; McIlfatrick et al., 2014; Walters et al., 2012). Others addressed lifestyle risks mainly in response to doctor referral or patient request (Beishuizen et al., 2019; James et al., 2020b; Jansink et al., 2010; Phillips et al., 2014). While nurses generally perceived lifestyle change as a personal responsibility (Bräutigam Ewe et al., 2021; Jansink et al., 2010; Phillips et al., 2014), they also described a role in strengthening patients' motivation (Bräutigam Ewe et al., 2021). As James et al. (2021) found, autonomous roles and organizational support facilitated nurses' participation and confidence in this area. There was potential to further develop GPN roles and enable nurses to work to their full scope of practice (Bräutigam Ewe et al., 2021; Goodman et al., 2011; Keleher & Parker, 2013). However, financial incentives and organizational priorities frequently influenced nursing activities (Hornsten et al., 2014; McIlfatrick et al., 2014; Walters et al., 2012). Resistance to role expansion plus poor interprofessional and intersectoral collaboration were common constraints (Geense et al., 2013; James et al., 2020b; Jansink et al., 2010; Keleher & Parker, 2013). General practice nurses called for greater organizational support and prioritization of prevention, more collaborative interdisciplinary relationships plus interprofessional training and professional development to optimize their role and advance their practice (Bräutigam Ewe et al., 2021; Geense et al., 2013; Goodman et al., 2011; James et al., 2020a; James et al., 2021; Jansink et al., 2010; Keleher & Parker, 2013; McIlfatrick et al., 2014; Walters et al., 2012).

### 3.4 Engaging mutual motivation

Joint investment and mutual motivation determined the outcomes of interactions and were necessary to encourage patient commitment to lifestyle risk reduction (Bräutigam Ewe et al., 2021; Geense et al., 2013; Hornsten et al., 2014; James et al., 2020b; Phillips et al., 2014). While patient awareness, readiness and willingness to change were common prerequisites for risk reduction, nurses' enthusiasm, motivation and confidence to support lifestyle risk reduction were key catalysts (Geense et al., 2013; James et al., 2021; Jansink et al., 2010; Keleher & Parker, 2013; Phillips et al., 2014). As Bräutigam Ewe et al. (2021) discovered, some GPNs saw motivating patients, particularly those resistant to lifestyle change, as tiring and difficult work. Others found it professionally rewarding when small successes increased patient motivation and commitment to sustain change.

A new diagnosis or threat of illness sometimes created teachable moments and stimulated patient's motivation to change (Hornsten et al., 2014; James et al., 2020b; Phillips et al., 2014). To increase the relevance of risks and patients' readiness for change, nurses linked risk communication to current or emerging health issues and symptoms (Bräutigam Ewe et al., 2021; Geense et al., 2013; Phillips et al., 2014). Severity of risk and potential health complications were also stressed to increase the urgency and relevance of risk reduction and to provide extrinsic motivation to modify lifestyle behaviour (Hornsten et al., 2014; Jansink et al., 2010; Keleher & Parker, 2013; Phillips et al., 2014). When assessing risk, nurses asked patients about their current health and lifestyle behaviours (Goodman et al., 2011; Hornsten et al., 2014; James et al., 2020a; McIlfatrick et al., 2014), some explored underlying reasons for behaviours (Geense et al., 2013) and discussed more immediate benefits of lifestyle change (James et al., 2020a; Phillips et al., 2014).

Communication strategies, based on motivational interviewing and behaviour change techniques, were used to explore patients' motivation and confidence to change, resolve ambivalence, address barriers and preempt setbacks (Beishuizen et al., 2019; Bräutigam Ewe et al., 2021; Geense et al., 2013; Hornsten et al., 2014; James et al., 2020b; McIlfatrick et al., 2014; Phillips et al., 2014; Tong et al., 2021). Nurses using behaviour change techniques most often provided ‘feedback on outcomes of behaviour’, ‘reviewed behaviour goal(s),’ and engaged in ‘problem solving’ (Tong et al., 2021; Westland et al., 2018). While some nurses were confident to motivate patients (Beishuizen et al., 2019; Goodman et al., 2011; McIlfatrick et al., 2014; Phillips et al., 2014), observational studies showed general practice nurses often did not assess patients' self‐confidence, explore barriers to change or identify strategies to overcome them (James et al., 2020a; Westland et al., 2018). Tong et al. (2021) similarly noted that, despite longer consultation times, nurses' use of behaviour change techniques was infrequent and variable. Reciprocally low motivation, negative attitudes and experiences and poor results were frustrating, causing some nurses to limit their involvement in lifestyle risk reduction (Aranda & McGreevy, 2014; Geense et al., 2013; Hornsten et al., 2014; James et al., 2020b; Jansink et al., 2010; McIlfatrick et al., 2014; Phillips et al., 2014).

### 3.5 Enabling confident action

Once motivated, patients were sometimes described as holding unrealistic expectations and lacking confidence and capacity to overcome obstacles to change (Beishuizen et al., 2019; Phillips et al., 2014). Therefore, nurses needed to negotiate realistic, actionable goals that were linked to patients' priorities (Beishuizen et al., 2019; Hornsten et al., 2014; James et al., 2021; Jansink et al., 2010; Phillips et al., 2014). Continuing conversations were necessary to attain a level of health literacy and risk awareness that stimulated readiness to change (Bräutigam Ewe et al., 2021; James et al., 2020b; Phillips et al., 2014). Providing personalized information, advice and education were considered essential nursing skills that increased patients' knowledge and ability to reduce lifestyle risks (Beishuizen et al., 2019; Hornsten et al., 2014; James et al., 2020a; James et al., 2021; Keleher & Parker, 2013). In routine consultations, lifestyle advice was often related to diet and physical activity (Geense et al., 2013; Phillips et al., 2014; Tong et al., 2021; Westland et al., 2018). Tong et al. (2021), for example, observed that portion control and encouragement of walking were the most common dietary and physical activity recommendations provided. Nevertheless, many GPNs felt they lacked the time, knowledge, skills and confidence to provide effective lifestyle education (Bräutigam Ewe et al., 2021; Geense et al., 2013; Goodman et al., 2011; James et al., 2021; Jansink et al., 2010; Keleher & Parker, 2013; Phillips et al., 2014), and opportunities were sometimes missed (Hornsten et al., 2014; James et al., 2020a; McIlfatrick et al., 2014; Tong et al., 2021). James et al. (2021) highlighted that educational preparation, continuing professional development and confidence affected the level of nurses' engagement and many expressed a desire to develop their knowledge and skills. As one small study showed, nurses' confidence to support goal setting improved following training in health coaching methods. Nevertheless, their efforts were limited due to a lack of follow‐up training and organizational support (Walters et al., 2012).

Several papers described enablement strategies including goal setting, action planning, monitoring patients' progress and referring them to other providers for additional support (Bräutigam Ewe et al., 2021; Geense et al., 2013; Goodman et al., 2011; Hornsten et al., 2014; James et al., 2020a; Phillips et al., 2014; Westland et al., 2018). Nurses reported that devising achievable, goal‐focused plans that detailed measurable actions and outcomes, and enabled monitoring and follow‐up, while effective, was also time and skill intensive (Goodman et al., 2011; James et al., 2020a; Phillips et al., 2014; Walters et al., 2012). Many nurses were not confident with these processes (James et al., 2020a; Jansink et al., 2010; Walters et al., 2012). When negotiating actions, small changes increasing in intensity over time were usually suggested. Outcomes were evaluated in terms of negotiated targets (Goodman et al., 2011; Phillips et al., 2014). Few papers described nurses advocating the use of self‐monitoring tools to enhance self‐management (Beishuizen et al., 2019; Phillips et al., 2014; Westland et al., 2018).

Regular follow‐up encouraged adherence and motivation while also enabling monitoring of progress and support for patients experiencing difficulties (Beishuizen et al., 2019; Phillips et al., 2014). Referrals to general practitioners, allied health providers and local community services were usually arranged (Goodman et al., 2011; Hornsten et al., 2014; James et al., 2020a; Phillips et al., 2014). However, nurses reported issues with referral pathways and lack of suitable, affordable and available local services (Bräutigam Ewe et al., 2021; Geense et al., 2013; Goodman et al., 2011; Jansink et al., 2010). While opportunities existed for general practice nurses to take a greater role in arranging recalls, follow‐ups and multidisciplinary care (Keleher & Parker, 2013), time pressures, workload, siloed practice and deficiencies in organizational capacity, management and funding were persistent barriers (Bräutigam Ewe et al., 2021; Hornsten et al., 2014; James et al., 2020a; James et al., 2021; Jansink et al., 2010; Keleher & Parker, 2013; Walters et al., 2012; Westland et al., 2018).

## 4 DISCUSSION

Familiar, trusting relationships create a safe environment that enables GPNs to deepen their knowledge of patients' preferences and priorities, raise risk awareness and health literacy, and promote self‐determination and self‐efficacy (Young et al., 2017). Meeting basic human needs for connection, safety and compassion, Soklaridis et al. (2016) showed that relationship‐centred care can improve the quality and efficacy of risk reduction and behaviour change interventions. While the literature links empathy, self and social awareness, emotional presence and mindfulness with greater relational satisfaction, cooperation and motivation (Kozlowski et al., 2017), this review demonstrated significant variability in GPNs' interpersonal skills. Practice and experience were found to increase relational sensitivity and empathy. As Minster (2020) and Kozlowski et al. (2017) suggest, training, role‐modelling and mentoring are needed to cultivate and develop these competencies. Future research should seek to understand the effectiveness of such strategies in building GPNs' capacity for relationship‐centred care.

Patient empowerment, engagement and enablement are synergistic processes and outcomes of shared decision‐making (WHO, 2021). As patients acquire greater influence, motivation and ability, they are better able to actively participate with health professionals to make informed decisions about their health and care options (Lambrinou et al., 2019). Flannery (2017) reveals that enhancing autonomy, competence and relatedness increased motivation whereas authoritative approaches increased patients' resistance to change. By acknowledging emotions, providing choice and encouraging participation while minimizing efforts to induce, direct and instruct, GPNs promote patients' independence, intrinsic motivation, confidence and ability to self‐determine and attain their health goals (Desborough et al., 2018; Flannery, 2017; Young et al., 2017). However, this review showed GPNs' use of participatory approaches was inconsistent. Resonating with Flannery (2017) and Vallis et al. (2018), nurses in this review believed they respected patients' autonomy and endeavoured to involve them in decision‐making. However, they recognized that they also tended to engage in communication that involved the use of advise, direct, instruct and control type strategies. Kozlowski et al. (2017) confirm that authoritative, pessimistic and dismissive attitudes, lack of confidence in interventions, and low priority for risk reduction are often related. This presents opportunities to enhance future practice through education that builds and maintains GPNs' motivation, communication skills and confidence with participatory approaches (Vallis et al., 2018; WHO, 2021).

Similarly, while motivational interviewing enabled GPNs to assess patients' risk awareness, readiness and confidence to reduce risks (James et al., 2018; Lambrinou et al., 2019), these skills were generally underdeveloped and underutilized. As James et al. (2018) indicated, ongoing training and support are needed to maintain proficiency in these complex skills. Likewise, although nurses strove to tailor information, health education and interdisciplinary support to promote patients' health literacy, self‐efficacy and independence (Desborough et al., 2018; Vallis et al., 2018), they lacked confidence with care planning and referral pathways. Similar to Young et al. (2017), findings in this review suggest that health mentoring and behaviour change counselling methods can provide GPNs with the philosophical foundations, knowledge and sophisticated interpersonal skills needed to support shared decision‐making. Further research should explore GPNs' ability to access training programs and peer supports and evaluate the impact of these methods on GPN knowledge and skill as well as patient satisfaction and health outcomes.

The availability of nurses in general practice, nurses' confidence in the value and efficacy of risk reduction interventions, collaboration with enthusiastic colleagues and organizations that prioritized risk reduction and optimized GPNs' roles were potent facilitators. Nevertheless, current funding structures, management practices and organizational cultures continue to restrict GPNs' role, scope and autonomy of practice, reinforcing task‐focused practice (Halcomb & Ashley, 2017). As Desborough et al. (2018) and James et al. (2018) recommend, issues related to GPNs' role and training, time, funding and organizational support must be addressed to optimize future clinical practice about lifestyle risk reduction. Despite the reported acceptability and effectiveness of nurse‐led lifestyle risk reduction, evidence remains unclear and research elucidating the successful elements of such interventions is needed (Stephen et al., 2022).

### 4.1 Limitations

Despite its contribution, the relatively small number of studies and the narrow geographic distribution of papers retrieved and included in this review may be a limitation. Broader inclusion criteria were considered, however the volume of results and emphasis on interventions rather than interactions made this strategy unsuitable. Additionally, the grey literature was not included due to the lack of peer review and rigour in reporting. While providers in other primary care settings also support lifestyle risk reduction, continued expansion of nursing in general practice justified the focus of this paper. Although other nursing professionals may also play a role in risk reduction, this review focused on the role of registered nurses who form the majority of the GPN workforce.

## 5 CONCLUSION

This review corroborates evidence for relationship‐centred care and reinforces the important role of GPNs in cultivating collaborative relationships that promote shared decision‐making, readiness for change, health literacy and enhanced capacity for risk reduction. Findings confirm synergistic relationships between patient participation, motivation and confidence and nurses' attitudes toward patients and interventions as well as their interpersonal, risk communication and care planning skills. As previous research has shown, lack of prioritization, time, training, funding, interprofessional collaboration and organizational support must be addressed to enhance GPNs' roles and motivation, and to equip them with the knowledge, skills and resources they need to effectively support lifestyle risk reduction. This review also highlights a gap in understanding synergistic processes involved in lifestyle risk reduction support provided by nurses in general practice. Further research may provide a theoretical conceptualisation of the process and inform strategies that strengthen GPNs' involvement, competence and confidence in the area of lifestyle risk reduction.

## AUTHOR CONTRIBUTIONS

All authors have agreed on the final version and meet at least one of the following criteria (recommended by the ICMJE*): (1) substantial contributions to conception and design, acquisition of data or analysis and interpretation of data; (2) drafting the article or revising it critically for important intellectual content.

## FUNDING INFORMATION

MM has been supported by an Australian Government Research Training Program Doctoral scholarship.

## CONFLICT OF INTEREST

No conflict of interest has been declared by the authors.

## Supporting information


Appendix S1

## Data Availability

Data sharing not applicable to this article as no datasets were generated or analysed during the current study
