# Supplementary material for: Understanding how general practice nurses support adult lifestyle risk reduction: An integrative review
Source: J Adv Nurs. 2022 Jun 30;78(11):3517–30. doi: 10.1111/jan.15344 (PMC9796034; doi:10.1111/jan.15344)
Supplement: Supplementary file 1 — Appendix S1 [file JAN-78-3517-s001.docx]

| **Integrated Literature Review – Mixed Methods Appraisal Tool (MMAT 2018)** | | | | | | | | | | | | | | | | | | | | | | | | | |
| --- | --- | --- | --- | --- | --- | --- | --- | --- | --- | --- | --- | --- | --- | --- | --- | --- | --- | --- | --- | --- | --- | --- | --- | --- | --- |
|  |  | |  | |  | | | | | | | | | | | | | | | | | | | | |
|  | | Aranda and McGreevy (2014) | | | | Beishuizen et al. (2019) | Brautigam Ewe  (2021) | Geense  et al. (2013) | Goodman et al. (2011) | Hornsten  et al. (2014) | James  et al. (2020a) | James  et at. (2020b) | James et al. (2021) | Jansink  et al. (2010) | Keleher  and Parker (2013) | | McIlfatrick et al. (2014) | | Phillips, Wood, and Kinnersley (2014) | Tong  et al. (2021) | | Walters  et al. (2012) | | Westland et al. (2018) | |
| **Qualitative** | | | | | |  |  |  |  |  |  |  |  |  | |  | |  |  | |  | |  | |  |
| Is the approach suitable to answer research aims? | | | | Yes | | Yes | Yes | Yes |  | Yes | Yes | Yes | Yes | Yes | | Yes | | Yes | Yes | |  | | Yes | |  |
| Are data collection methods adequate to address research aims? | | | | Yes | | Yes | Yes | Yes |  | Yes | Yes | Yes | Yes | Yes | | No | | Yes | Yes | |  | | Yes | |  |
| Are findings adequately derived from data? | | | | Yes | | Yes | Yes | Yes |  | Yes | Yes | Yes | Yes | Yes | | Yes | | Yes | Yes | |  | | Yes | |  |
| Does data substantiate interpretation of results? | | | | Yes | | Yes | Yes | Yes |  | Yes | Yes | Yes | Yes | Yes | | Yes | | Yes | Yes | |  | | Yes | |  |
| Is there cohesion between data sources, collection, analysis & interpretation? | | | | Yes | | Yes | Yes | Yes |  | Yes | Yes | Yes | Yes | Yes | | Yes | | Yes | Yes | |  | | Yes | |  |
| **Quantitative** | | | | | |  |  |  |  |  |  |  |  |  | |  | |  |  | |  | |  | |  |
| Does the sampling strategy  address research aims? | | | | | |  |  |  | Yes |  |  |  |  |  | |  | | Yes |  | | Yes | | Yes | | Yes |
| Is the sample representative  of the target population? | | | | | |  |  |  | Can't  tell |  |  |  |  |  | |  | | Can’t  tell |  | | Yes | | No | | No |
| Are measurements appropriate? | | | | | |  |  |  | Yes |  |  |  |  |  | |  | | Yes |  | | Yes | | Yes | | Yes |
| Is risk of nonresponse bias low? | | | | | |  |  |  | Can't  tell |  |  |  |  |  | |  | | No |  | | Can’t tell | | No | | No |
| Is statistical analysis appropriate  to answer the research aim? | | | | | |  |  |  | Yes |  |  |  |  |  | |  | | Yes |  | | Yes | | Yes | | Yes |
| **Mixed Methods** | | | | | |  |  |  |  |  |  |  |  |  | |  | |  |  | |  | |  | |  |
| Is the rationale for using mixed methods to address the research aim adequate? | | | | | | |  |  |  |  |  |  |  |  | |  | | Yes |  | |  | | Yes | |  |
| Are different components integrated  to answer the research aim? | | | | | | |  |  |  |  |  |  |  |  | |  | | Yes |  | |  | | Yes | |  |
| Are integrated outputs  adequately interpreted? | | | | | |  |  |  |  |  |  |  |  |  | |  | | Yes |  | |  | | Yes | |  |
| Are inconsistencies & divergences between results addressed? | | | | | |  |  |  |  |  |  |  |  |  | |  | | Yes |  | |  | | Yes | |  |
| Do different components adhere to their traditional quality criteria? | | | | | |  |  |  |  |  |  |  |  |  | |  | | Yes |  | |  | | Yes | |  |
